# Clinical and Immunologic Impact of CMV Coinfection Among Children Living With HIV in Canada

**DOI:** 10.1097/INF.0000000000004811

**Published:** 2025-04-07

**Authors:** Yves Fougère, Jason Brophy, Michael T. Hawkes, Terry Lee, Lindy Samson, Soren Gantt, Mi-Suk Kang Dufour, Christian Renaud, Hinatea Dieumegard, Madeleine Aby Diallo, Jade Canape, Stanley Read, Ari Bitnun, Hugo Soudeyns, Fatima Kakkar

**Affiliations:** From the * Unit of Pediatric Infectious Diseases and Vaccinology, Department of Woman Mother and Child, Lausanne University Hospital, Lausanne, Switzerland; † Centre d’infectiologie mère-enfant (CIME), Centre de recherche Azrieli du CHU Sainte-Justine, Montreal, Quebec, Canada; ‡ Children’s Hospital of Eastern Ontario, University of Ottawa, Ottawa, Ontario, Canada; § BC Children’s Hospital and Department of Pediatrics, University of British Columbia, Vancouver, British Columbia, Canada; ¶ CIHR Pan-Canadian Network for HIV & STBBI Clinical Trials Research (CTN+), Vancouver, British Columbia, Canada; ∥ Department of Microbiology, Infectiology & Immunology; ** Department of Pediatrics, Faculty of Medicine, Université de Montréal, Montreal, Quebec, Canada; †† University of California, Berkeley, School of Public Health; ‡‡ Unité d’immunopathologie virale, Centre de recherche Azrieli du CHU Sainte-Justine, Montreal, Quebec, Canada; §§ Department of Pediatrics, The Hospital for Sick Children, University of Toronto, Toronto, Ontario, Canada; ¶¶ Department of Pathology and Laboratory Medicine, Emory University School of Medicine, Atlanta, Georgia, USA.; British Columbia (BC) Women’s Hospital & Health Centre, Vancouver; Centre de recherche du Centre hospitalier de l’Université de Montréal, Department of Microbiology, Infectiology & Immunology, Université de Montréal; Hospital for Sick Children, Department of Pediatrics, University of Toronto; Children’s Hospital of Eastern Ontario, Department of Pediatrics, University of Ottawa; Children’s Hospital of Winnipeg, University of Manitoba; National Institute of Allergy and Infectious Diseases, Bethesda; University of British Columbia, Vancouver; Children’s Hospital of Winnipeg, University of Manitoba; BC Children’s Hospital and Department of Pediatrics, University of British Columbia, Vancouver; Centre hospitalier universitaire (CHU) Sainte-Justine, Department of Pediatrics, Université de Montréal; Montreal Children’s Hospital, Department of Pediatrics, McGill University; University Health Network, Department of Medicine, University of Toronto; National Human Immunodeficiency Virus (HIV) and Retrovirology Laboratory (NHRL), Public Health Agency of Canada (PHAC), Winnipeg; CHU Sainte-Justine, Department of Pediatrics, Université de Montréal; CHU Sainte-Justine, Department of Pediatrics, Université de Montréal; BC Women’s & Children’s Hospital, Vancouver; Canadian Institutes of Health Research (CIHR) Canadian HIV Trials Network (CTN), Vancouver; BC Women’s Hospital & Health Centre, University of British Columbia, Vancouver; Montreal Children’s Hospital, Department of Pediatrics, McGill University; Hospital for Sick Children, Department of Pediatrics, University of Toronto; Public/Global Health Consultant, San Francisco; Children’s Hospital of Eastern Ontario, Department of Pediatrics, University of Ottawa; NHRL, PHAC, Winnipeg; BC Women’s Hospital & Health Centre, Department of Pediatrics, University of British Columbia, Vancouver; McMaster Children’s Hospital, Department of Pediatrics, McMaster University, Hamilton; CIHR CTN, Vancouver; Centre de recherche Azrieli du CHU Sainte-Justine, Department of Microbiology, Infectiology & Immunology and Department of Pediatrics, Université de Montréal; Department of Pediatrics, University of Saskatchewan, Saskatoon; Stollery Children’s Hospital, Department of Pediatrics, University of Alberta, Edmonton

**Keywords:** HIV, cytomegalovirus, coinfection, children, T cell subsets

## Abstract

**Background:**

Although cytomegalovirus (CMV) disease has been well described among severely immunocompromised children living with HIV (CLWH), the impact of CMV coinfection, is not well understood. The objective of this study was to characterize the clinical and immunologic effects of CMV coinfection in CLWH in Canada.

**Methods:**

This is a substudy of the Early Pediatric Initiation, Canada Child Cure Cohort study, which enrolled CLWH in Canada between 2014 and 2018. CMV serostatus was determined at the first (baseline) study visit, and HIV-1 viral load (VL), CMV VL, and lymphocyte subsets were quantified every 3–6 months. For a subset of participants, CD4^+^ and CD8^+^ T cell subsets were analyzed using flow cytometry. The clinical outcomes were recorded retrospectively at the baseline visit and prospectively during the study period.

**Results:**

Of the 225 participants, 85.3% were CMV seropositive (CMV^+^) and 81% had suppressed HIV VL. While there were no significant differences in clinical outcomes between CMV^+^ and CMV^−^ children, CMV^+^ children had lower frequencies of CD4^+^ T cells, higher frequencies of CD8^+^ T cells, and lower CD4/CD8 ratio at baseline than CMV^−^ children. Children with CMV^+^ children also demonstrated a higher frequency of CD4^+^ effector memory cells, lower CD8^+^ naïve T cells, and higher frequencies of CD8^+^ terminally differentiated effector memory cells. These differences remained significant even after adjusting for HIV viral control.

**Conclusions:**

CMV coinfection is common among CLWH and is associated with distinct immunological changes despite the effective control of HIV replication with antiretroviral therapy. The long-term implications of these immunologic perturbations require further investigation.

Although cytomegalovirus (CMV) coinfection is common among both adults and children living with HIV (CLWH),^1–3^ the long-term impact of CMV coinfection among people living with HIV (PLWH) remains unclear. Multiple manifestations of CMV-associated disease in immunocompromised individuals are well described, with clear guidelines for diagnosis, treatment, and follow-up.^4–6^ However, the impact of CMV coinfection alone, as evidenced by positive CMV IgG serostatus or episodes of CMV viremia in the absence of overt CMV^−^ associated disease, is less clear. While current North American guidelines recommend against routine testing for CMV serostatus among asymptomatic adults living with HIV,^7^ testing is recommended for all CLWH after a year of age, as is screening for congenital CMV infection in all infants with vertically transmitted HIV.^6^ However, there are no recommendations for the routine assessment of CMV viremia in asymptomatic adults or children known to be CMV seropositive (CMV^+^).

Of all herpesviruses, CMV has the most dynamic interaction with the human immune system and the greatest potential to affect the immune system of PLWH. In healthy individuals, CMV infection elicits a high frequency of CMV-specific T cells that engage in a lifelong battle to restrain CMV replication and prevent associated diseases. In CMV-seropositive individuals, approximately 10% of circulating CD4^+^ and CD8^+^ memory T cells are CMV-specific,^8^ which can increase to 30% of CD4^+^ T cells and 50% of CD8^+^ T cells in older adults, with impaired expansion of naïve T cells and an inverted CD4/CD8 ratio.^3^ Although antiretroviral therapy (ART) with sustained viral suppression generally improves the CD4/CD8 ratio and allows for relative normalization of the distribution of T cell subsets, CMV coinfection and replication in PLWH could potentially impair this process and explain persistent immune dysfunction in individuals with well-controlled HIV infection.^9,10^

Most CLWH acquires CMV in the first year of life, when their developing immune system is already challenged with the control of perinatal HIV infection.^2^ While a limited number of studies, predominantly from resource-limited settings, have demonstrated that CMV coinfection in CLWH is associated with more severe clinical outcomes,^11,12^ the impact of CMV coinfection in the absence of the disease is less known.^13,14^ Historically, neither CMV serostatus nor CMV replication has been routinely assessed in asymptomatic CLWH due to a lack of evidence for intervention (eg, valganciclovir treatment). Given the recent availability of newer, less toxic anti-CMV antivirals (eg, letermovir and maribavir) and the ongoing efforts to develop a CMV vaccine, a better understanding of the impact of chronic CMV infection in CLWH may inform new recommendations. Therefore, the objective of this study was to characterize the clinical and immunological effects of CMV coinfection in CLWH in Canada.

## METHODS

### Study Design

This was a substudy of the prospective, multicenter Early Pediatric Initiation, Canada Child Cure Cohort study (EPIC4), which was designed to evaluate the dynamics of the HIV reservoir^14^ and chronic HIV inflammation^15^ among children with perinatally acquired HIV-1 infection. EPIC^4^ enrolled CLWH from 8 major clinical centers across Canada between December 2014 and December 2018. Children with nonperinatal HIV infection were excluded. Children received their medical care under a system of universal healthcare, and the standard of care for HIV treatment in all centers followed the US Department of Health and Human Services guidelines for the treatment of pediatric HIV infection at the time.^16^ The study visits (every 3–6 months) were concurrent with routine clinical visits. Voluntary informed consent was provided by the participants or their legal guardians as appropriate. The EPIC^4^ cohort and its substudies were approved by the Research Ethics Boards of all the participating institutions.

Children were enrolled beginning in December 2014, and the first visit was defined as the “baseline visit.” At every visit, whole blood, serum and plasma were collected, and peripheral blood mononuclear cells (PBMCs) were isolated. At the baseline visit, a comprehensive review of the child’s clinical history, treatment history and all available laboratory measures (from birth to baseline visit) was recorded through chart review. These data were then collected prospectively throughout the study period and entered the REDCap database hosted by the CIHR Canadian HIV Trials Network (CTN, Vancouver, British Columbia).

### CMV Serostatus

Titers of CMV-specific IgG and IgM were determined at baseline for all children using the Architect chemiluminescent microparticle immunoassay (Abbott Diagnostics, Mississauga, ON).^17–19^ Children were considered CMV^+^ if serum IgG titer was ≥6.0 AU per mL of serum^17^ or if serum IgM titer was ≥1.00 AU per mL of serum at baseline.^19^ For those who were CMV^+^, IgG avidity testing was performed to document the timing of infection, with high avidity (≥60%) being suggestive of an infection >4 months prior. CMV serology was repeated at the end of follow-up in those who were seronegative at baseline and in seropositive children under age 2 years to account for passive transfer of maternal antibodies.

### CMV Viremia

CMV DNA viral load (VL) in plasma was quantified at every visit using an AltoStar AM16 automated system and the AltoStar CMV PCR Kit 1.5 (Altona Diagnostics GmbH, Hamburg, Germany) according to the manufacturer’s instructions. The lower limit of detection for this assay was 215 IU/mL of plasma (95% confidence interval: 163–330 IU/mL of plasma).^20^ CMV viremia was defined as the presence of any detectable CMV VL at least once during follow-up.

### HIV-1 Viremia

HIV VL was measured at every visit per the routine standard of care. The techniques used to measure the levels of HIV-1 RNA in plasma and lower limits of detection varied between study sites (target not detected or <20 or <40 HIV-1 RNA copies per mL of plasma). HIV-1 viremia during follow-up was defined as the presence of VL at or above the assay cutoff for detectable virus according to the assay used.

### Immunophenotyping

PBMCs were thawed, washed and stained to measure the expression of cell surface markers using multiparameter flow cytometry. Cell viability ranged between 38.0% and 85.9% (median = 75.1%). Cells were stained using PerCP-conjugated anti-CD3 monoclonal antibody (mAb; UCHT1 clone), BUV737-conjugated anti-CD4 mAb (SK3 clone), PE-CF594-conjugated anti-CCR7 mAb (150503 clone), BV650-conjugated anti-CD45RA mAb (HI100 clone), APC-H7-conjugated anti-CD8 mAb (SK1 clone) and Live/Dead AF700 (Invitrogen, Waltham, MA). All mAbs were sourced from BD Biosciences (Mississauga, Ontario, Canada). A median of 1.70 × 10^6^ live PBMCs (range = 306,553–6.43 × 10^6^) was acquired on a BD Fortessa flow cytometer using FACSDiva software v9.0 (BD Biosciences). The lymphocyte gate was set using forward and side scatter. Antibody capture beads (UltraComp eBeads, Invitrogen) were used for single color compensation control for each mAb. Gates were defined using fluorescence minus one controls using sample from a healthy donor. The gating strategy is depicted in Figure, Supplemental Digital Content 1, http://links.lww.com/INF/G156.

CD4^+^ and CD8^+^ T cells subsets were defined as follows: CD4^+^ naïve (CD4^+^ T_N_; CD4^+^CD45RA^+^CCR7^+^), CD4^+^ central memory (CD4^+^ T_CM_; CD4^+^CD45RA^−^CCR7^+^), CD4^+^ effector memory (CD4^+^ T_EM_; CD4^+^CD45RA^−^CCR7^−^), CD8^+^ naïve (CD8^+^ T_N_; CD8^+^CD45RA^+^CCR7^+^), CD8^+^ central memory (CD8^+^ T_CM_; CD8^+^CD45RA^−^CCR7^+^), CD8^+^ effector memory (CD8^+^ T_EM_; CD8^+^CD45RA^−^CCR7^−^) and CD8^+^ terminally differentiated effector memory (CD8^+^ T_EMRA_; CD8^+^CD45RA^+^CCR7^−^).^21^ The CD4/CD8 ratio was calculated by dividing the absolute number of CD4^+^ cells by the absolute number of CD8^+^ cells.

### Clinical Outcomes

The following outcomes were identified by retrospective chart review, both at the time of entry into the cohort (baseline), and in longitudinal clinical follow-up over the study period (follow-up): (1) lifetime hospitalization (defined as any hospitalization over the lifetime); (2) history of CMV disease (including retinitis, colitis, central nervous system disease, hepatitis, pneumonitis and thrombocytopenia over the lifetime); (3) coinfections, including physician-diagnosed history of symptomatic herpes simplex virus infection (herpes labialis or stomatitis), active tuberculosis, meningococcal or pneumococcal disease over the lifetime; (4) neurodevelopmental delay (as entered by physicians in the medical record) and (5) worst lifetime Centers for Disease Control and Prevention (CDC) clinical staging, defined using the CDC criteria.^22^

### Statistical Analysis

General, clinical and lymphocyte subset characteristics were summarized using proportions for categorical variables and medians with interquartile ranges (IQR) for continuous variables. Comparisons of characteristics by CMV serostatus and occurrence of CMV viremia were assessed using the chi-square test for categorical variables and Wilcoxon rank-sum tests for continuous outcomes. Unadjusted linear regressions were used to estimate the associations between lymphocyte or T cell subsets and CMV serostatus or CMV viremia. A multivariable linear regression model was then constructed, adjusting for variables identified a priori as potential confounders (age at baseline, age at treatment initiation, presence of concurrent HIV viremia, occurrence of any ART interruption during the study and migration status). Baseline characteristics that were highly dependent on the group definition were not included to avoid overadjustment. Among those with CMV viremia, a linear generalized estimation model was constructed to compare T cell subsets at the time of CMV viremia versus no viremia, to account for the repeated measures within individuals. Statistical analyses were performed using R software and the R Studio interface, version 2022.02.3 (R Studio Inc.).

## RESULTS

### Baseline Characteristics

Among 228 children enrolled in the EPIC^4^ cohort between December 2014 and December 2018, 225 were included in the substudy (2 withdrew their consent, and 1 had insufficient samples for CMV testing). The baseline demographics and HIV treatment details are presented in Table 1. The median age at baseline was 13.8 years (IQR = 9.3–17.0; range = 0.4–26.4 years), and the median duration of follow-up was 32 months (IQR = 21–38 months; range = 0–42 months), with a median number of visits of 8 (IQR = 5–12; range = 1–16). Nearly all (98.7%) children received ART at baseline. Overall, 81% of the patients had viral suppression at baseline, and 73% maintained suppression for the entire duration of follow-up.

**TABLE 1.** General Characteristics of Participants and Clinical Outcomes According to CMV Serostatus

Characteristics	All Participants (n = 225)	CMV Seropositive (n = 192)	CMV Seronegative (n = 33)	*P* Value
General				
Female, n (%)	121 (53.8)	101 (52.6)	20 (60.6)	0.51
Median age in years, (IQR)	13.8 (9.3–17.0)	13.9 (9.3–17.0)	13.6 (9.0–17.5)	0.72
Immigrated to Canada, n (%)	126 (56.0)	120 (62.5)	6 (18.2)	**<0.001**
Receiving ART at baseline, n (%)	222(98.7)	189 (97.9)	33 (100)	0.90
Viral suppression at baseline, n (%)	182 (81)	154 (79.8)	28 (84.8)	0.66
Median age in years at ART initiation (IQR)*	3.2 (0.5–7.7)	3.7 (0.9–7.9)	1.0 (0.2–3.6)	**0.005**
No cART during study, n (%)	22(9.8)	18 (9.4)	4 (12.1)	0.86
At least one episode of HIV viremia during study, n (%)	61 (27.1)	53 (27.6)	8 (24.2)	0.94
CDC HIV Infection stage at baseline Stage 3 CD4 % age-specific, n (%) Stage 3 CD4 count age-specific, n (%)	6 (2.7) 6 (2.7)	6 (3.1) 6 (3.1)	0 (0) 0 (0)	0.657 0.657
Clinical outcomes†				
Any hospitalization, n (%)	135 (60.0)	115 (59.9)	20 (60.6)	1
CMV disease‡, n (%)	8 (3.6)	8 (4.1)	0 (0.0)	0.50
HSV disease, n (%)	19 (8.4)	16 (8.3)	3 (9.1)	1
Active tuberculosis, n (%)	21 (9.3)	20 (10.4)	1 (3.0)	0.31
Any pneumococcal/meningococcal infection, n (%)	8 (3.6)	5 (2.6)	3 (9.1)	0.17
Neurodevelopmental delay, n (%)	43 (19.1)	38 (19.8)	5 (15.2)	0.699
Worst lifetime CDC clinical stage, n (%) A/N B C	122 (54.2) 52 (23.1) 51 (22.7)	106 (55.2) 43 (22.4) 43 (22.4)	16 (48.5) 9 (27.3) 8 (24.2)	0.598 0.696 0.993

*P*-values < 0.05 are highlighted in bold.

* Eight missing data in the CMV-seropositive group.

† Clinical outcome data is cumulative, and includes baseline data (all data until entry into the cohort) and all follow-up time points for the duration of study).

‡ Eight participants had CMV disease including 1 retinitis, 2 colitis, 2 hepatitis and 5 pneumonitis.

### CMV Serostatus

Of the 192 (85.3%) participants who were CMV^+^ at baseline, 186 (96.8%) demonstrated high avidity for CMV IgG. Only 1 of the CMV^−^ participants at baseline seroconverted to CMV^+^ by the end of the follow-up and no CMV^+^ child <2 years of age reverted to CMV^−^ status. While there was no difference in age or sex among CMV^+^ versus CMV^−^ children, children who were CMV^+^ were significantly more likely to be born outside Canada (62.5 vs. 18.2%, *P* < 0.001) and older at the age of ART initiation (3.7 vs. 1.0 years, *P* = 0.005). Among the CMV^+^ children, 8 (4.1%) had a documented history of CMV disease associated with immunosuppression (pneumonitis, colitis, hepatitis or retinitis) (Table 1), all of which occurred before the baseline visit. There were no significant differences in the clinical outcomes between CMV^+^ and CMV^−^ children (Table 1).

### Distribution of CD4^+^ and CD8^+^ T Cells According to CMV Serostatus

**FIGURE 1.**
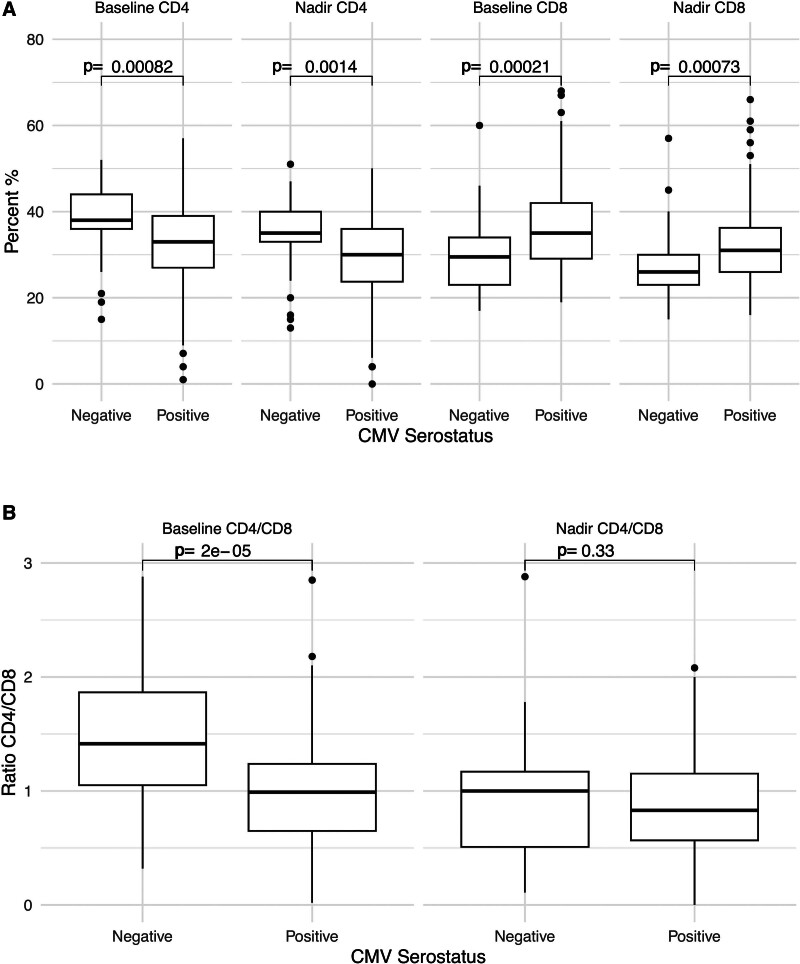
Distribution of CD4^+^ and CD8^+^ T cells according to CMV serostatus. A: Relative baseline and nadir frequencies of CD4^+^ and CD8^+^ T cells according to CMV serostatus. B: Baseline and nadir CD4/CD8 ratios according to CMV serostatus.

Figure 1 shows the distribution of frequencies of CD4^+^ and CD8^+^ T cells and the CD4/CD8 ratio among CLWH according to CMV serostatus at baseline (first visit) and study nadir for the duration of follow-up. At baseline, CLWH who were CMV^+^ had significantly higher CD8^+^ T cell counts (757.5 vs. 586 cells/µL, *P* = 0.019; Figure, Supplemental Digital Content 2, http://links.lww.com/INF/G157), significantly lower CD4^+^ T cell frequency (33% vs. 38%, *P* < 0.001; Fig. 1A), significantly higher CD8^+^ T cell frequency (35% vs. 29.5%, *P* < 0.001; Fig. 1A), and significantly lower CD4/CD8 ratio (0.99 vs. 1.42, *P* < 0.001; Fig. 1B) compared with CMV^−^ children. Absolute CD4^+^ T cell counts were similar between CMV^+^ and CMV^−^ CLWH at baseline (672 vs. 770 cells/µL, *P* = 0.12). Children who were CMV^+^ also had significantly higher nadir CD8^+^ T cell counts (550 vs. 449 cells/µL, *P* = 0.021; Figure, Supplemental Digital Content 2, http://links.lww.com/INF/G157), significantly lower nadir CD4^+^ T cell frequency (30% vs. 35%, *P* = 0.001), significantly higher nadir CD8^+^ T cell frequency (31.0% vs. 26.0%, *P* < 0.001), and lower nadir CD4/CD8 ratio (0.83 vs. 1.00, *P* = 0.33) compared with CMV^−^ children (Fig. 1A, B). Except for CD8^+^ T cell counts at baseline, all these differences remained significant on both the univariate and multivariate regression, after adjusting for age, age at ART initiation, detection of HIV viremia during the study period, migration status and occurrence of any ART interruption during the study period (Table 2A).

**TABLE 2.** Association Between Lymphocytes Subset or Lymphocytes T Subsets and CMV

Variables	Univariate Analyses			Multivariable Analyses		
	Slope	95% CI	*P* Value	Slope	95% CI	*P* Adjusted
A.Association between lymphocytes subsets and positive CMV serostatus (N = 192)						
Baseline						
Absolute CD4^+^ T cell count	−150.1	−307.1 to 6.9	0.06	−94.8	−242.5 to 52.9	0.21
CD4^+^ T cell frequency (%)	−5.3	− 8.7 to −2.0	**0.002**	−3.9	− 7.1 to −0.6	**0.020**
Absolute CD8^+^ T cell count	123.1	−13.2 to 259.4	0.08	122.3	−16.7 to 261.3	0.08
CD8^+^ T cell frequency (%)	6.7	3.0 to 10.4	**<0.001**	6.3	2.8 to 9.8	**<0.001**
CD4/CD8 ratio	−0.4	−0.6 to −0.2	**<0.001**	−0.3	−0.5 to −0.2	**<0.001**
Nadir values during follow-up						
Absolute CD4^+^ T cell count	−59.2	−170.3 to 52.0	0.30	−16.9	−120.0 to 86.2	0.75
CD4^+^ T cell frequency (%)	−5.4	−8.9 to −1.9	**0.003**	−4.5	−7.8 to −1.3	**0.006**
Absolute CD8^+^ T cell count	105.3	4.5 to 206.2	**0.041**	122.3	16.2 to 228.4	**0.024**
CD8^+^ T cell frequency (%)	4.8	1.6 to 8.0	**0.003**	5.2	2.1 to 8.2	**<0.001**
CD4/CD8 ratio	−0.1	−0.3 to 0.1	0.25	−0.1	−0.3 to 0.1	0.37
B.Association between T lymphocyte subsets and positive CMV serostatus (N = 52)						
CD4^+^ T cell subsets						
CD4^+^ T cell frequency (%)	−5.6	−12.6 to 1.5	0.12	−4.1	−11.0 to 2.8	0.24
CD4^+^ T_N_ frequency (%)	−6.1	−14.2 to 2.0	0.14	−9.6	−17.4 to −1.9	**0.015**
CD4^+^ T_CM_ frequency (%)	1.5	−4.4 to 7.4	0.61	3.3	−2.5 to 9.1	0.25
CD4^+^ T_EM_ frequency (%)	2.8	−0.1 to 5.6	0.06	3.1	0.1 to 6.2	**0.044**
CD8^+^ T cell subsets						
CD8^+^ T cell frequency (%)	4.9	−1.8 to 11.6	0.15	3.0	−3.6 to 9.6	0.37
CD8^+^ T_N_ frequency (%)	−15.8	−26.1 to −5.5	**0.003**	−17.4	−28.1 to −6.8	**0.002**
CD8^+^ T_CM_ frequency (%)	−0.1	−1.3 to 1.1	0.82	0.1	−1.2 to 1.3	0.88
CD8^+^ T_EM_ frequency (%)	3.5	0.2 to 6.9	**0.040**	2.77	−0.9 to 6.4	0.14
CD8^+^ T_EMRA_ frequency (%)	9.2	2.2 to 16.3	**0.011**	10.9	3.4 to 18.4	**0.005**

*P*-values < 0.05 are highlighted in bold.

Multivariable analysis: Adjusted for HIV viremia during the study, age, age at treatment initiation, any treatment interruption during the study and migration status.

CI indicates confidence interval.

### Distribution of T Cell Subsets

The distribution of CD4^+^ and CD8^+^ naïve memory-effector T cell subsets was assessed using flow cytometry in a subgroup (n = 65) of study participants (Table 3). No differences in total, T_N_ or T_CM_ cell frequencies were observed in CD4^+^ T cells between CMV^+^ and CMV^−^ participants; however, CMV^+^ participants had a significantly higher frequency of CD4^+^ T_EM_ cells (5.7% vs. 3.7%, *P* = 0.005). While the total frequencies of total CD8^+^ T cells and CD8^+^ T_CM_ cells were similar among CMV^+^ and CMV^−^ children (39.7% vs. 31.8% and 2.5% vs. 2.9%, respectively), CMV^+^ children exhibited significantly lower frequencies of CD8^+^ T_N_ cells (54.6 vs. 70.9%, *P* = 0.003) and significantly higher frequencies of CD8^+^ T_EM_ cells (6.8 vs. 4.6%, *P* = 0.047) and CD8^+^ T_EMRA_ cells (19.5 vs. 9.6%, *P* = 0.003). Apart from CD8 T_EM_ cells, all these differences remained significant on both the univariate and multivariate regression analysis, after adjusting for age, age at ART initiation, detection of HIV viremia during the study period, migration status and occurrence of any ART interruption during the study period (Table 2B).

**TABLE 3.** T Cell Subsets According to CMV Serostatus

T cell Subsets	Participants With T Cell Subsets Available N = 65	CMV Seropositive N = 52	CMV Seronegative N = 13	*P* Value
General characteristics				
Female, n (%)	34 (52.3)	27 (51.9)	7 (53.8)	1
Median age in years (IQR)	14.4 (10.0–17.0)	14.4 (9.8–17.5)	14.8(12.6–17.5)	0.54
Immigrated to Canada, n (%)	29 (44.6)	28 (53.8)	1 (7.7)	0.007
Receiving ART at baseline, n (%)	63 (96.9)	50 (96.2)	13 (100)	1
Viral suppression at baseline, n (%)	53 (81.5)	40 (76.9)	13 (100)	0.13
Median age in years at ART initiation (IQR)*	2.5 (1.0–9.0)	3.4 (0.8–9.2)	1.2 (0.4–3.6)	0.35
Any treatment interruption during study, n (%)	6 (9.2)	5 (9.6)	1 (7.7)	1
At least one episode of HIV viremia during study, n (%)	26 1.17 (40)	24 (46.2)	2 (15.4)	0.09
CD4^+^ T cell subsets				
CD4^+^ T cell frequency, % (IQR)	55.0 (47.0–61.0)	52.5 (47.0–59.8)	59.2 (54.7–62.6)	0.09
CD4^+^ T_N_ frequency, % (IQR)	66.4 (55.0–74.0)	63.4(54.2–73.6)	73.6 (69.3–74.9)	0.133
CD4^+^ T_CM_ frequency, % (IQR)	19.5 (15.0–28.0	20.9 (15.0–28.1)	16.7 (15.3–20.2)	0.54
CD4^+^ T_EM_ frequency, % (IQR)	5.0 (4.0–7.0)	5.7 (4.0–7.5)	3.7 (3.3–4.0)	**0.005**
CD8 subsets				
CD8^+^ T cell frequency, % (IQR)	38.5 (32.0–45.0)	39.7 (33.3–47.7)	31.8 (29.6–38.9)	0.11
CD8^+^ T_N_ frequency, % (IQR)	57.1 (44.0–66.0)	54.6(40.7–63.3)	70.9(57.6–77.4)	**0.003**
CD8^+^ T_CM_ frequency, % (IQR)	2.5 (2.0–4.0)	2.5 (1.7–4.1)	2.9 (2.2–3.3)	0.92
CD8^+^ T_EM_ frequency, % (IQR)	6.1 (4.0–10.0)	6.8 (3.8–11.0)	4.6 (2.1–6.6)	**0.047**
CD8^+^ T_EMRA_ frequency, % (IQR)	17.2 (10.0–25.0)	19.5(10.8–27.4)	9.6(6.8–12.9)	**0.003**

*P*-values < 0.05 are highlighted in bold.

* Missing data: 3 missing data in CMV seropositive group.

### CMV Viremia

Thirty-four (17.7%) of the CMV^+^ children had CMV viremia detected at least once during the follow-up (median CMV VL among detectable VL, 422 IU/mL; range = 85–1991 IU/mL). Of these, 25 (74%) were CMV viremic only once, 4 (12%) twice, and 5 (15%) had ≥3 episodes of CMV viremia during the study period. Children who had at least one episode of CMV viremia were more likely to be younger (12.7 vs. 14.3 years, *P* = 0.048), to be born outside versus within Canada (79.4% vs. 58.9%, *P* = 0.04), and to have had at least one episode of HIV viremia during the study period (70.6% vs. 36.4%, *P* < 0.001) (Table, Supplemental Digital Content 3, http://links.lww.com/INF/G158). None of the CMV patients had detectable CMV viremia during follow-up. Overall, there was no significant difference in CD4^+^ T cell frequencies or CD4/CD8 ratios at baseline and the study nadir among those with and without CMV viremia during the study period, although those who were CMV viremic had a higher frequency of CD8^+^ T cells at baseline (38 vs. 35%, *P* = 0.033) (Table, Supplemental Digital Content 3, http://links.lww.com/INF/G158). This remained significant after adjusting for age, age at treatment initiation, presence of concurrent HIV viremia, occurrence of any treatment interruption during the study, and migration status (Table, Supplemental Digital Content 4, http://links.lww.com/INF/G159). Among those with CMV viremia, there was no significant difference in CD4 or CD8^+^ T cell frequencies, or CD4/CD8 ratios, at the time of viremia versus not.

## DISCUSSION

This is one of the largest studies to assess the impact of CMV coinfection on clinical and immunological outcomes in CLWH. In this well-characterized pediatric cohort with detailed VL measures of HIV and CMV over time, we found that CMV coinfection was common among CLWH in Canada, with over 85% seropositivity at a median age of 14 years, and nearly 1/5 experiencing at least one episode of CMV viremia during follow-up. While neither CMV serostatus nor the occurrence of CMV viremia were shown to have an impact on clinical outcomes in this study, marked changes in immune profiles were observed in children according to CMV serostatus. CMV^+^ children had significantly lower frequencies of CD4^+^ T cells, higher frequencies of CD8^+^ T cells, and lower CD4/CD8 ratios at baseline than their CMV^−^ counterparts. These differences remained significant after adjusting for potential cofounders, suggesting that CMV coinfection, independent of HIV control, may drive these changes. This is consistent with previously reported data in adults living with HIV, where CMV serostatus has been associated with immunological changes in individuals with well-controlled HIV infections.^9,23,24^ In a study of adults treated with ART for a minimum of 2 years, CMV serostatus was independently associated with a CD4/CD8 ratio <1.^9^ In a separate study of adults initiated on ART, normalization of the CD4/CD8 ratio was delayed among CMV coinfected individuals, potentially due to an expansion of the CD8^+^ T cell compartment.^23^ However, data on children are limited. In 1 cohort of infants with perinatally acquired HIV infection, those with CMV coinfection had significantly lower CD4^+^ T cell counts and frequencies and significantly higher absolute CD8^+^ T cell counts and frequencies at different time points during follow-up compared with participants who were CMV uninfected.^12^ In another study of ART-experienced CLWH with severe HIV disease who initiated a new ART regimen at baseline, those with CMV coinfection had a slower recovery of CD8^+^ T_N_ and a higher frequency of T_EMRA_ during a follow-up period of 40 weeks.^13^ In our study, CMV^+^ children exhibited significantly lower frequencies of CD8^+^ T_N_ cells and significantly higher frequencies of CD8^+^ T_EM_ cells and CD8^+^ T_EMRA_. While there were no differences in total T_N_ or T_CM_ cell frequencies, CMV^+^ children had a significantly higher frequency of CD4^+^ T_EM_ cells, with differences that remained statistically significant in multivariate models. Taken together, these findings indicate that the response to CMV is associated with the activation of CD8^+^ T cells and the subsequent depletion of CD8^+^ T_N_ cells in the setting of HIV coinfection. These results suggest that CMV coinfection may alter the differentiation and maturation of CD4^+^ and CD8^+^ T cells in CLWH independent of HIV viral suppression and timing of ART initiation.

In this cohort of well-treated children on ART, 17.7% of CMV^+^ patients had CMV viremia during the 4-year follow-up period. This is significantly lower than the previously reported frequencies of CMV viremia with untreated HIV infection, which ranged from 26.3% to 54% in adults and children, respectively,^10,11,25^ indicating that control of HIV replication may reduce the incidence of CMV viremia. The incidence of asymptomatic CMV viremia in CLHIV on ART is not well known. In one cohort of children in Zimbabwe,^10^ CMV viremia was detected in 38.4% of CLWH who were ART-naïve, 24.0% of CLWH on ART, and 8.9% of children without HIV infection. The association between the occurrence of CMV and HIV viremia has also been observed in other pediatric cohorts,^11,26^ and suggests that ART-mediated HIV virologic suppression affects the occurrence of CMV viremia.

The long-term impacts of immunologic changes observed in CMV^+^ CLWH remain to be determined. Absolute CD4^+^ T cell count was not associated with CMV coinfection among CLWH in this cohort, though CD4% and CD4/CD8 ratios were lower. Absolute numbers and relative frequencies of T cells are both of biological importance and are interrelated. For example, lower CD4^+^ T cell frequencies could result from expansion of CD8^+^ T cells resulting from an ongoing CD8-mediated response against CMV. There is increasing evidence that lower CD4/CD8 ratios may be associated with increased long-term morbidity and mortality, potentially driven by immune activation, chronic inflammation and persistence of the HIV reservoir.^26^ The potential role of anti-CMV treatment with medications such as valganciclovir, letermovir or maribavir in reducing CMV-associated complications in PLWH remains to be determined.^27,28^ Finally, should a CMV vaccine become available, investigating its potential benefits in PLWH would be a high priority.^29^

Our study had several limitations. First, EPIC^4^ was a heterogeneous cohort of children with perinatal HIV infection who were of different ages and initiated ART at different ages. Although these differences were adjusted for in the analysis, there may have been residual confounders we were unable to adjust for. For example, the precise timing of CMV acquisition, the duration of HIV suppression and the virologic status at ART initiation, as well as other coinfections, could not be adjusted for. These factors might have contributed to the immunological changes observed. CMV-specific T cell function was not assessed in this study; therefore, it is not clear whether the T cell findings are due to CMV-specific T cells or bystander (non-CMV) T cells. Second, most subjects (85%) were CMV^+^, resulting in a much smaller sample size for the CMV^−^ group, which may have affected the results. Third, the duration of clinical follow-up was too short to observe the long-term impact of CMV on clinical events, which generally present with advanced age and late adulthood. Fourth, in the absence of a control group of children living without HIV, it is unknown whether the immunological findings reported in this cohort simply reflect an independent contribution of CMV infection, irrespective of HIV infection coinfection status. Finally, the duration between visits (3–6 months) may have been too long to capture all intermittent viremia, resulting in an underestimation of the true incidence of CMV viremia. Nonetheless, given the magnitude of the differences observed, our findings suggest a role for CMV coinfection, although we may have been limited in our conclusions on the role of CMV viremia.

## CONCLUSION

Among Canadian CLWH with well-controlled HIV infection, CMV coinfection is common and is associated with changes in the immunological profile of coinfected children, with lower CD4^+^ T cell frequencies, higher CD8^+^ T cell frequencies, a lower CD4/CD8 ratio, and remodeling of the T cell differentiation profile. Further research is necessary to clarify the impact of CMV on immune function and dysregulation in CLWH, and assessing inflammatory and specific CMV-T cell responses in this population could provide valuable insights. Finally, a longer duration of follow-up may be necessary to evaluate the potential effects of CMV coinfection on long-term clinical outcomes.

## ACKNOWLEDGMENTS

*The authors thank all study participants and the following individuals for their expertise and technical assistance: Cheryl Arneson, Christine Bon, Jennifer Bowes, Martine Caty, Anika Gerois, Zoe Hassall, Cathy den Hollander, Gillian Morantz, Audrée Janelle-Montcalm, Danny Dong Hyun Kim, Mbaye Ndiaye, Barb Neufeld, Laura Puri, Annie Qiu, Suzanne Taillefer and Silvie Valois. EPIC*^*4*^ *was a study of the Canadian HIV Trials Network (CTN-281) and was supported by the Canadian Institutes of Health Research (CIHR), the International AIDS Society (IAS), and the Canadian Foundation for AIDS Research (CANFAR) (grant no. HIG-133051). H.S. received an infrastructure grant from Réseau SIDA et MI of Fonds de la recherche du Québec-santé (FRQS). F.K. was the recipient of a Junior 2 Career Scholarship from the FRQS. Y.F. was the recipient of 2 Swiss fellowship grants from the Société académique vaudoise and the SICPA foundation.*

*The Early Pediatric Initiation Canada Child Cure Cohort (EPIC* ^*4*^ *) Study Group: Ariane Alimenti, British Columbia (BC) Women’s Hospital & Health Centre, Vancouver; Petronela Ancuta, Centre de recherche du Centre hospitalier de l’Université de Montréal, Department of Microbiology, Infectiology & Immunology, Université de Montréal; Ari Bitnun, Hospital for Sick Children, Department of Pediatrics, University of Toronto; Jason Brophy, Children’s Hospital of Eastern Ontario, Department of Pediatrics, University of Ottawa; Jared Bullard, Children’s Hospital of Winnipeg, University of Manitoba; Tae-Wook Chun, National Institute of Allergy and Infectious Diseases, Bethesda; Hélène C. F. Côté, University of British Columbia, Vancouver; Joanne Embree, Children’s Hospital of Winnipeg, University of Manitoba; Michael T. Hawkes, BC Children’s Hospital and Department of Pediatrics, University of British Columbia, Vancouver; Fatima Kakkar, Centre hospitalier universitaire (CHU) Sainte-Justine, Department of Pediatrics, Université de Montréal; Christos Karatzios, Montreal Children’s Hospital, Department of Pediatrics, McGill University; Rupert Kaul, University Health Network, Department of Medicine, University of Toronto; John Kim, National Human Immunodeficiency Virus (HIV) and Retrovirology Laboratory (NHRL), Public Health Agency of Canada (PHAC), Winnipeg; Valérie Lamarre, CHU Sainte-Justine, Department of Pediatrics, Université de Montréal; Normand Lapointe, CHU Sainte-Justine, Department of Pediatrics, Université de Montréal; Pascal Lavoie, BC Women’s & Children’s Hospital, Vancouver; Terry Lee, Canadian Institutes of Health Research (CIHR) Canadian HIV Trials Network (CTN), Vancouver; Deborah M. Money, BC Women’s Hospital & Health Centre, University of British Columbia, Vancouver; Dorothy Moore, Montreal Children’s Hospital, Department of Pediatrics, McGill University; Stanley Read, Hospital for Sick Children, Department of Pediatrics, University of Toronto; Robert Reinhard, Public/Global Health Consultant, San Francisco; Lindy Samson, Children’s Hospital of Eastern Ontario, Department of Pediatrics, University of Ottawa; Paul Sandstorm, NHRL, PHAC, Winnipeg; Laura Sauve, BC Women’s Hospital & Health Centre, Department of Pediatrics, University of British Columbia, Vancouver; Sandra Seigel, McMaster Children’s Hospital, Department of Pediatrics, McMaster University, Hamilton; Joel Singer, CIHR CTN, Vancouver; Hugo Soudeyns, Centre de recherche Azrieli du CHU Sainte-Justine, Department of Microbiology, Infectiology & Immunology and Department of Pediatrics, Université de Montréal; Ben Tan, Department of Pediatrics, University of Saskatchewan, Saskatoon; and Wendy Vaudry, Stollery Children’s Hospital, Department of Pediatrics, University of Alberta, Edmonton.*

## Supplementary Material



## References

[1] Gianella S Letendre S. Cytomegalovirus and HIV: a dangerous Pas de Deux. J Infect Dis. 2016;214(Suppl 2):S67–S74. 27625433 10.1093/infdis/jiw217 PMC5021239

[2] Slyker JA. Cytomegalovirus and paediatric HIV infection. J Virus Erad. 2016;2:208–214. 27781102 10.1016/S2055-6640(20)30873-6 PMC5075347

[3] Freeman ML Lederman MM Gianella S. Partners in crime: the role of CMV in immune dysregulation and clinical outcome during HIV infection. Physiol Behav. 2016;13:10–19. 10.1007/s11904-016-0297-9 PMC5079703 26810437

[4] NIH, Centers for Disease Control and Prevention, HIV Medicine Association of the IDSA. Guidelines for the prevention and treatment of opportunistic infections in adults and adolescents with HIV. 2023. Available at: https://clinicalinfo.hiv.gov/en/guidelines/hiv-clinical-guidelines-adult-and-adolescent-opportunistic-infections/whats-new. Accessed January 20, 2025.

[5] Nelson M Dockrell D Edwards S; BHIVA Guidelines Subcommittee. British HIV Association and British Infection Association guidelines for the treatment of opportunistic infection in HIV-seropositive individuals 2011. HIV Med. 2011;12:1–140. Available at: https://www.bhiva.org/file/SwhaEzgXmAGOt/hiv_v12_is2_Iss2Press_Text.pdf. 10.1111/j.1468-1293.2011.00944_1.x 21851517

[6] Panel on Opportunistic Infections in Children with and Exposed to HIV. Guidelines for the prevention and treatment of opportunistic infections in children with and exposed to HIV. Department of Health and Human Services. 2024. Available at: https://clinicalinfo.hiv.gov/en/guidelines/pediatric-opportunistic-infection. Accessed January 20, 2025.

[7] Thompson MA Horberg MA Agwu AL. Primary care guidance for persons with human immunodeficiency virus: 2020 update by the HIV Medicine Association of the Infectious Diseases Society of America. Clin Infect Dis. 2021;73:e3572–e3605. 33225349 10.1093/cid/ciaa1391

[8] Sylwester AW Mitchell BL Edgar JB. Broadly targeted human cytomegalovirus-specific CD4+ and CD8+ T cells dominate the memory compartments of exposed subjects. J Exp Med. 2005;202:673–685. 16147978 10.1084/jem.20050882 PMC2212883

[9] Caby F Guihot A Lambert-Niclot S. Determinants of a low CD4/CD8 ratio in HIV-1-infected individuals despite long-term viral suppression. Clin Infect Dis. 2016;62:1297–1303. 26908792 10.1093/cid/ciw076

[10] Yindom LM Simms V Majonga ED. Unexpectedly high prevalence of cytomegalovirus DNAemia in older children and adolescents with perinatally acquired human immunodeficiency virus infection. Clin Infect Dis. 2019;69:580–587. 30828710 10.1093/cid/ciy961 PMC6669294

[11] Wamalwa D Njuguna I Maleche-Obimbo E. Cytomegalovirus viremia and clinical outcomes in kenyan children diagnosed with human immunodeficiency virus (HIV) in hospital. Clin Infect Dis. 2022;74:1237–1246. 34214163 10.1093/cid/ciab604 PMC8994579

[12] Kovacs A M.D. Schluchter M Ph.D. Easley K M.S.. Cytomegalovirus infection and HIV-1 disease progression in infants born to HIV-1–infected women. Pediatric Pulmonary and Cardiovascular Complications of Vertically Transmitted HIV Infection Study Group. N Engl J Med. 1999;341:77–84. 10395631 10.1056/NEJM199907083410203 PMC4280563

[13] Kapetanovic S Aaron L Montepiedra G; for the Pediatric AIDS Clinical Trials Group Protocol 366. Effect of cytomegalovirus co-infection on normalization of selected T-cell subsets in children with perinatally acquired HIV infection treated with combination antiretroviral therapy. PLoS One. 2015;10:e0120474–e0120415. 25794163 10.1371/journal.pone.0120474 PMC4368806

[14] Bitnun A Ransy DG Brophy J; Early Pediatric Initiation Canada Child Cure Cohort (EPIC4) Research Group. Clinical correlates of human immunodeficiency virus-1 (HIV-1) DNA and inducible HIV-1 RNA reservoirs in peripheral blood in children with perinatally acquired HIV-1 infection with sustained virologic suppression for at least 5 years. Clin Infect Dis. 2020;70:859–866. 30919879 10.1093/cid/ciz251 PMC7319270

[15] Bernard I Ransy DG Brophy J; EPIC^4^ Study Group. Chitinase-3-like protein 1 is associated with poor virologic control and immune activation in children living with HIV. Viruses. 2022;14:2602–2612. 36560606 10.3390/v14122602 PMC9786985

[16] Panel on Antiretroviral Therapy and Medical Management of Children Living with HIV. Guidelines for the use of antiretroviral agents in pediatric HIV Infection. Department of Health and Human Services. 2024. Available at: https://clinicalinfo.hiv.gov/en/guidelines/pediatric-arv. Accessed January 20, 2025.

[17] Abbott. Architect CMV IgG. 2019. Available at: https://medilinkltd.com/wp-content/uploads/2023/07/CMV-IgG.pdf. Accessed March 20, 2025.

[18] Abbott. Architect CMV IgG Avidity. 2021. Available at: https://consultas.anvisa.gov.br/api/consulta/produtos/25351234875200810/anexo/T21924558/nomeArquivo/02.+IFU+3L46_ARCHITECT_CMV_IgG_Avidity_Rgt_IFU_G30041R07.pdf?Authorization=Guest. Accessed March 20, 2025..

[19] Abbott. Architect CMV IgM. 2019. Available at: https://medilinkltd.com/wp-content/uploads/2023/07/CMV-IgM.pdf. Accessed March 20, 2025..

[20] Altona Diagnostics. Instructions for Use AltoStar ® CMV RT-PCR Kit 1.5. 2021:1–106. Available at: https://altona-diagnostics.com/wp-content/uploads/2023/12/AS-CMV-1.5_MAN-AS0021540-EN-S01_WEB.pdf. Accessed March 20, 2025.

[21] Sallusto F Lenig D Förster R. Two subsets of memory T lymphocyteswith distinct homing potentials and effector functions. Nature. 1999;401:708–712. 10537110 10.1038/44385

[22] CDC. 1993 revised classification system for HIV infection and expanded surveillance case definition for AIDS among adolescents and adults. MMWR Recomm Rep. 1992;41(RR-17):1–19. 1361652

[23] Poizot-Martin I Allavena C Duvivier C; for the Dat’AIDS Study Group. CMV+ serostatus associates negatively with CD4:CD8 ratio normalization in controlled HIV-infected patients on cART. PLoS One. 2016;11:e0165774–e0165712. 27824907 10.1371/journal.pone.0165774 PMC5100980

[24] Freeman ML Mudd JC Shive CL. CD8 T-cell expansion and inflammation linked to CMV coinfection in ART-treated HIV infection. Clin Infect Dis. 2016;62:392–396. 26400999 10.1093/cid/civ840 PMC4706630

[25] Durier N Ananworanich J Apornpong T. Cytomegalovirus viremia in Thai HIV-infected patients on antiretroviral therapy: prevalence and associated mortality. Clin Infect Dis. 2013;57:147–155. 23511301 10.1093/cid/cit173

[26] Slyker JA Lohman-Payne BL John-Stewart GC. Acute cytomegalovirus infection in Kenyan HIV-infected infants. AIDS. 2009;23:2173–2181. 19617812 10.1097/QAD.0b013e32833016e8 PMC2761509

[27] Gianella S Kitch DW Beck-engeser G. Suppressing asymptomatic CMV with letermovir reshapes cardiometabolic proteome in treated HIV. In: Vol CROI 2024; 2024:Poster 354.

[28] Royston L Isnard S Berini CA. Influence of letermovir treatment on gut inflammation in people living with HIV on antiretroviral therapy: protocol of the open-label controlled randomised CIAO study. BMJ Open. 2023;13:e067640–e067649. 10.1136/bmjopen-2022-067640 PMC9872486 36690406

[29] Nelson CS Herold BC Permar SR. A new era in cytomegalovirus vaccinology: considerations for rational design of next-generation vaccines to prevent congenital cytomegalovirus infection. NPJ Vaccines. 2018;3:38. 30275984 10.1038/s41541-018-0074-4 PMC6148244

