# Supplementary figures and images for: Clinical and Immunologic Impact of CMV Coinfection Among Children Living With HIV in Canada
Source: Pediatr Infect Dis J. 2025 Apr 7;44(8):764–71. doi: 10.1097/INF.0000000000004811 (PMC12240138; doi:10.1097/INF.0000000000004811)

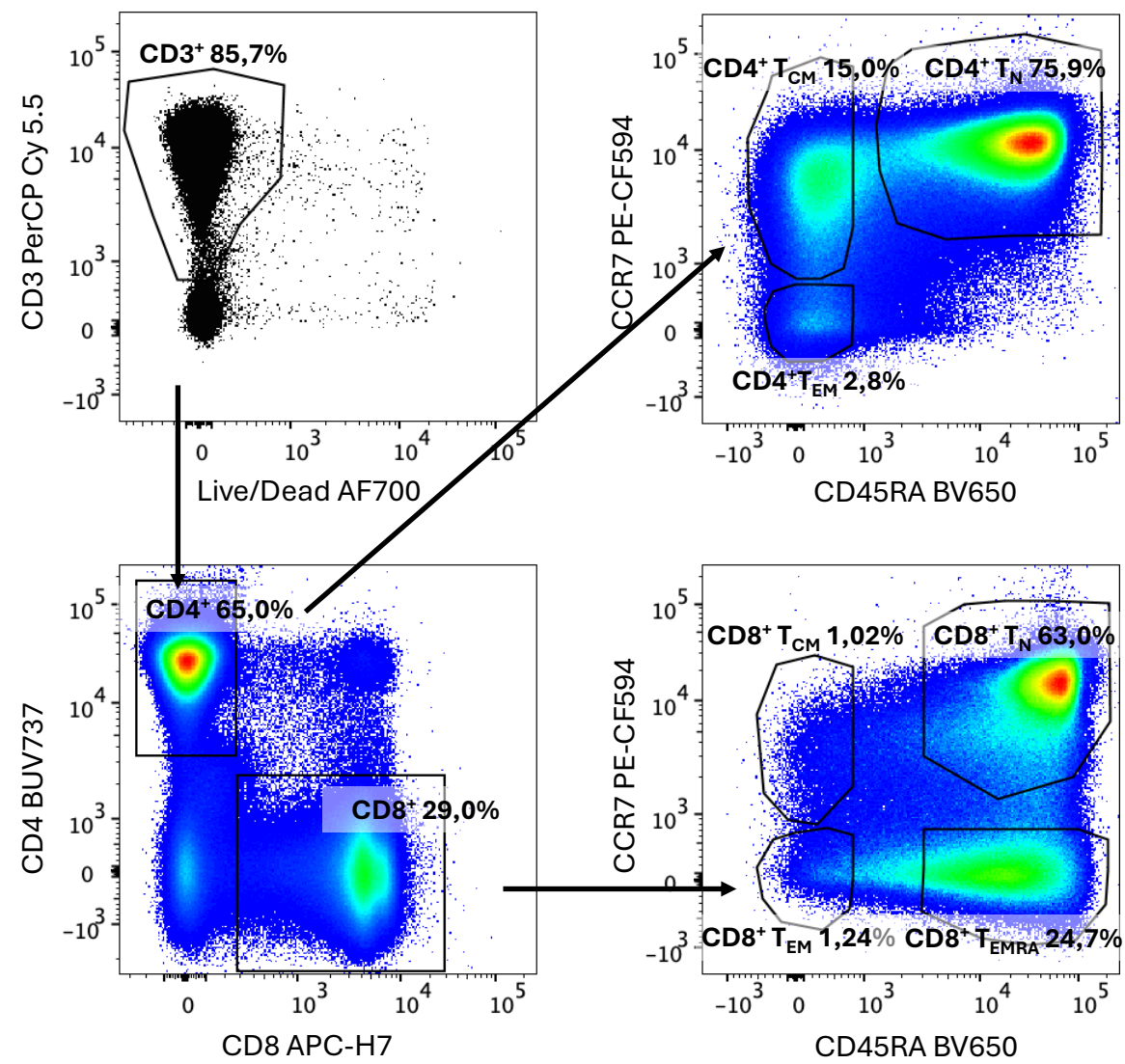

Supplement: Supplementary file 1 [file inf-44-0764-s001.pdf]
