# Supplementary material for: Clinical and Immunologic Impact of CMV Coinfection Among Children Living With HIV in Canada
Source: Pediatr Infect Dis J. 2025 Apr 7;44(8):764–71. doi: 10.1097/INF.0000000000004811 (PMC12240138; doi:10.1097/INF.0000000000004811)

SUPPLEMENTAL DIGITAL CONTENT 2. Absolute Baseline and Nadir CD4+ and CD8+ T Cell Counts According to CMV Serostatus.

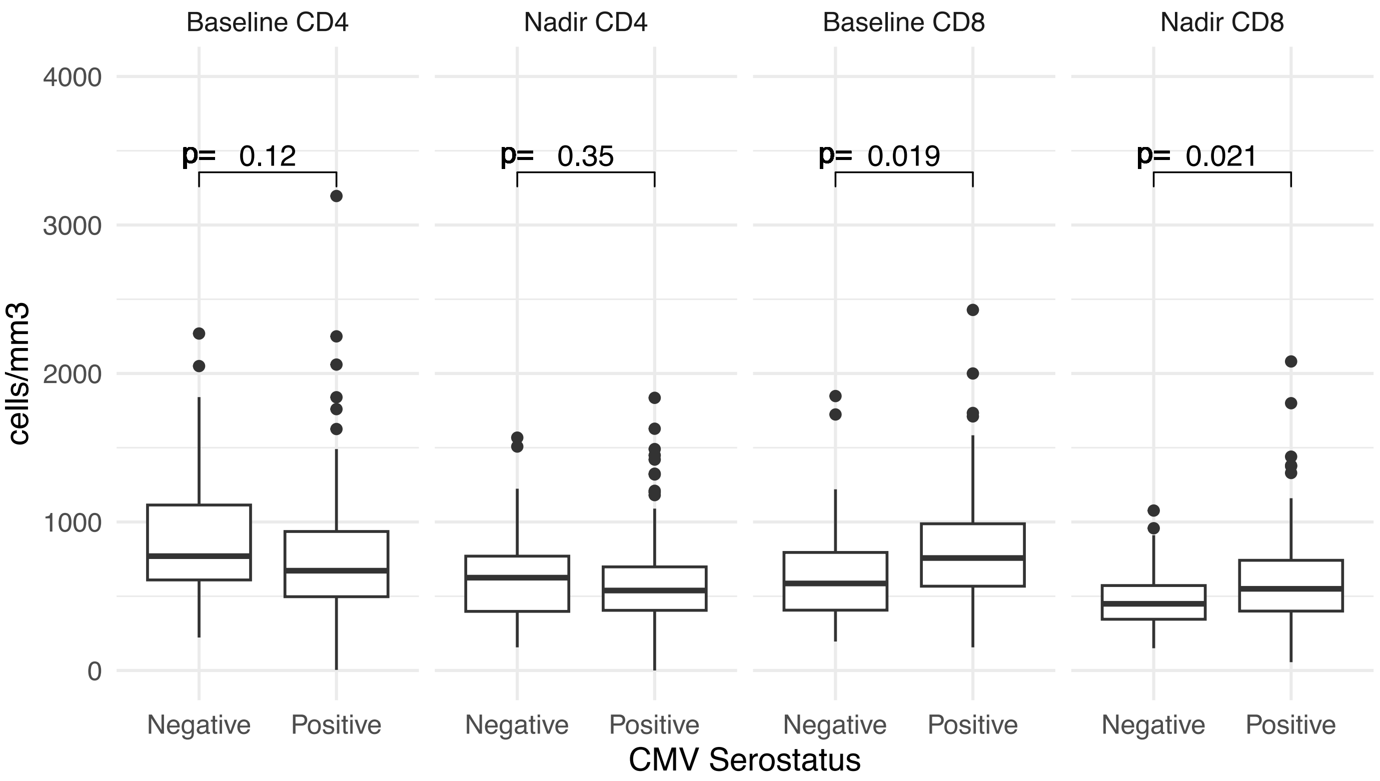

Supplement: Supplementary file 2 [file inf-44-0764-s002.pdf]
