# Supplementary material for: Clinical and Immunologic Impact of CMV Coinfection Among Children Living With HIV in Canada
Source: Pediatr Infect Dis J. 2025 Apr 7;44(8):764–71. doi: 10.1097/INF.0000000000004811 (PMC12240138; doi:10.1097/INF.0000000000004811)
Supplement: Supplementary file 3 [file inf-44-0764-s003.pdf]

SUPPLEMENTAL DIGITAL CONTENT 3. Clinical and immunologic outcomes according to CMV viremia

Characteristics	All participants N=192	Any CMV viremia N=34	No CMV Viremia n=158	P value
**General**				
Female, n (%)	101 (52,6)	18 (52.9)	83 (52,5)	1
Median age in years (IQR)	13.9 (9.3-17.0)	12.7 (7.4-16.2)	14.3 (10.4-17.2)	**0.048**
Immigrated to Canada, n (%)	120 (62.5)	27 (79.4)	93 (58.9)	**0.040**
Receiving ART at baseline, n (%)	189 (98.4)	33 (97.1)	156 (98.7)	1
HIV Viral suppression at baseline, n (%)	38 (19.8)	13 (38.2)	25 (15.8)	**0.006**
Median age at ART initiation *	3.7 (0.9-7.9)	4.4 (1.5-9.3)	3.7 (0.7-7.7)	0.12
Any ART interruption during study, n (%)	18 (9.4)	5 (14.7)	13 (8.2)	0.39
At least one episode of HIV viremia during study, n (%)	79 (41.1)	24 (70.6)	55 (34.8)	**<0.001**
**Clinical outcomes during study follow-up**				
Hospitalization during follow-up, n (%)	24 (12.5)	3 (8.8)	21 (13.3)	0.67
CMV disease during follow-up, n (%)	0 (0)	0 (0)	0 (0)	NA
**Lymphocytes subset at Baseline**				
CD4 count cells/µL (IQR)	672.0 (496.8-936.0)	702 (409.0 -1004.0)	666 (501.0-914.0)	0.93
CD4 % (IQR)	33.0 (27.0-39.0)	33.0 (23.0-39.0)	34.0 (28.0-39.0)	0.23
CD8 count cells/µL (IQR)	757.5 (567.5-988.0)	800.0 (621.0-1157.0)	755.0 (550.0-971.0)	0.15
CD8 % (IQR)	35.0 (29.1-42.0)	38.0(32.0-45.0)	35.0(28.0-42.0)	**0.033**
CD4/CD8 ratio (IQR)	1.0 (0.7-1.2)	0.9(0.5-1.1)	1.0(0.7-1.3)	0.10
**Lowest lymphocytes subset during study**				
CD4 count cells/µL (IQR)	538.5 (405.0-697.8)	529.5 (319.5-971.0)	540.0 (415.5-661.0)	0.83
CD4 % (IQR)	30.0 (23.8-36.0)	27.0 (19.3-35.8)	30.0 (24.0-36.0)	0.08
CD8 count cells/µL (IQR)	500.0 (400.0-742.0)	555 (447.8-753.0)	545.5 (394.3-740.0)	0.43
CD8 % (IQR)	31.0 (26.0-36.3)	30.0 (27.3-34.0)	31.0 (25.0-36.8)	0.81
CD4/CD8 ratio (IQR)	0.8 (0.6-1.2)	0.7 (0.5-1.0)	0.8 (0.6-1.1)	0.40

****Missing data:*** *8 total missing data, 2 in any CMV viremia group, 6 in no CMV viremia group.*
