# Supplementary material for: Clinical and Immunologic Impact of CMV Coinfection Among Children Living With HIV in Canada
Source: Pediatr Infect Dis J. 2025 Apr 7;44(8):764–71. doi: 10.1097/INF.0000000000004811 (PMC12240138; doi:10.1097/INF.0000000000004811)
Supplement: Supplementary file 4 [file inf-44-0764-s004.pdf]

SUPPLEMENTAL DIGITAL CONTENT 4. Association between Lymphocytes subset and CMV viremia during study

**Variables**	**Univariate analyses**			**Multivariable analyses**		
	**Slope**	**95% CI**	**P value**	**Slope**	**95% CI**	**P adjusted**
**Association between lymphocytes subset values at Baseline and CMV viremia**						
**CD4 count**	4.9	-147.5 to 157.4	0.95	- 43.8	-187.2 to 99.6	0.55
**CD4%**	-2.3	-5.7 to 1.1	0.19	- 1.2	-4.5 to 2.1	0.47
**CD8 count**	97.4	-37.4 to 232.3	0.16	34.8	-108.9 to 178.5	0.63
**CD8%**	4.2	0.5 to 8.0	**0.027**	3.9	0.4 to 7.5	**0.030**
**CD4/CD8 ratio**	-0.2	- 0.3 to 0.0	0.06	- 0.1	-0.3 to 0.0	0.12
**Association between lowest lymphocytes subset values during study and CMV viremia**						
**CD4 count nadir**	67.0	-41.6 to 175.6	0.23	79.7	-21.9 to 181.3	0.12
**CD4% nadir**	-3.4	-6.9 to 0.2	0.06	-0.9	-4.2 to 2.5	0.61
**CD8 count nadir**	45.5	-58.9 to 150.0	0.39	40.6	-73.8 to 154.9	0.49
**CD8% nadir**	1.2	-2.1 to 4.4	0.48	2.1	-1.0 to 5.2	0.18
**CD4/CD8 ratio nadir**	-0.1	-0.2 to 0.1	0.43	-0.1	-0.2 to 0.1	0.49

Multivariable analysis: Adjusted for HIV viremia during study, age, age at treatment initiation, any treatment interruption during study, and migration status.
